# Cardiovascular safety assessment of pramlintide in type 2 diabetes: results from a pooled analysis of five clinical trials

**DOI:** 10.1186/s40842-016-0030-z

**Published:** 2016-05-11

**Authors:** Kathrin Herrmann, Ming Zhou, Andrew Wang, Tjerk W. A. de Bruin

**Affiliations:** 1 Bristol-Myers Squibb/AstraZeneca, San Diego, CA USA; 2 Bristol-Myers Squibb, Hopewell, NJ USA; 3 AstraZeneca CVMD GMD, One Medimmune Way, Gaithersburg, MD 20878 USA

**Keywords:** Amylin, Cardiovascular safety, Insulin, Major adverse cardiovascular events, Pramlintide, Type 2 diabetes

## Abstract

**Background:**

This report evaluated the cardiovascular safety of the amylin analog pramlintide—an existing diabetes injectable treatment—by comparing relevant cardiovascular adverse events (AEs) reported in previous phase 3 and 4 clinical trials among patients receiving pramlintide and those receiving control treatments.

**Methods:**

Cardiovascular safety of pramlintide was assessed using accepted regulatory medical definitions of AEs reported in five randomized, controlled phase 3 and 4 trials of 16–52 weeks’ duration in adults with type 2 diabetes. The original trials compared pramlintide (90–120 mcg twice daily or 30–150 mcg three times daily) with placebo (four studies) or a mealtime rapid-acting insulin analog (one study). Background therapies included insulin alone or in combination with oral glucose-lowering agents. AE data obtained from clinical study reports were combined into one database and analyzed for the intention-to-treat population of 2016 patients (pramlintide, *n* = 1434; pooled comparator, *n* = 582). The primary analysis compared reported major adverse cardiovascular events (MACE) between pramlintide and control.

**Results:**

The incidence of reported MACE was similar between pramlintide (4.7 %) and pooled comparators (4.5 %). Secondary analyses included MACE relative risk and hazard ratio point estimates, which ranged from 0.86 to 0.93 for pramlintide relative to comparator treatment; the upper limit of the two-sided 95 % confidence interval did not exceed the threshold of 1.8.

**Conclusions:**

Both the point estimate of the reported MACE frequency and estimated risk ratios showed that mealtime pramlintide as an adjunct to insulin conferred no increased risk of cardiovascular AEs in patients with type 2 diabetes using insulin.

## Background

Diabetes mellitus is a complex disease that is progressive in nature and involves a disrupted interplay of glucoregulatory hormones. In normal physiology, insulin and amylin are co-secreted by pancreatic β cells in response to meals. Both insulin and amylin responses to food intake are altered in patients with type 2 diabetes and absent in patients with type 1 diabetes, because of autoimmune β-cell destruction [1, 2].

Pramlintide is a soluble analog of amylin, a neuroregulatory hormone that slows the rate of gastric emptying and decreases food intake in the long term by acting on the efferent vagal nerve [3, 4]. Additionally, pramlintide attenuates immediate postprandial glucagon secretion, which in turn can reduce hepatic glucose production [4, 5]. Combined, the mechanisms of action of pramlintide contribute to reduced glycemia following ingestion of exogenous glucose [2, 4, 6–8].

Pramlintide at doses of 30 and 60 mcg in patients with type 1 diabetes and at a dose of 120 mcg in patients with type 2 diabetes has been shown to result in plasma pramlintide concentrations that approximate physiological postprandial plasma amylin concentrations in healthy subjects [8]. Placebo-controlled clinical trials of pramlintide in type 1 diabetes [9–11] and type 2 diabetes [12–14] have shown that mealtime pramlintide injections as an adjunct to mealtime insulin significantly reduce glycated hemoglobin (HbA1c) levels (by 0.3–0.7 % and 0.3–1.0 %, respectively) and body weight (by 0.4–1.4 kg and 0.5–1.8 kg in type 1 and type 2 diabetes, respectively). The most commonly reported adverse events (AEs) were nausea, vomiting, anorexia or reduced appetite, and severe hypoglycemia in studies in patients with type 1 diabetes (when the insulin dose was not simultaneously reduced), and nausea and mild to moderate hypoglycemia in studies in patients with type 2 diabetes [9–12, 15, 16].

In this report, an evaluation of cardiovascular safety of pramlintide was conducted using accepted regulatory medical definitions of AEs in five previous randomized, controlled phase 3 and 4 trials of 16- to 52-weeks’ duration in adults with type 2 diabetes.

## Methods

### Study selection

This assessment analyzed data from an integrated database of all randomized controlled trials of pramlintide with a duration of 16 to 52 weeks conducted in patients with type 2 diabetes (Table 1). Cardiovascular safety of pramlintide—an existing injectable mealtime treatment for diabetes in adjunct with insulin—was assessed using accepted regulatory medical definitions of AEs reported in five randomized, controlled phase 3 and 4 trials of 16- to 52-weeks’ duration in adults with type 2 diabetes. The original trials compared pramlintide (90–120 mcg twice daily or 30, 60, 75, 120, or 150 mcg three times daily; Table 1) with placebo (four studies) or a mealtime rapid-acting insulin analog (one study) [12–16]. Background therapies included insulin alone or in combination with oral glucose-lowering agents. AE data were obtained from clinical study reports, which were combined into one database. The studies used in the present report are summarized in Table 1, and the demographics and patient characteristics are shown in Table 2. Cardiovascular events were not prospectively adjudicated. Clinical study reports were the source of the reports of cardiovascular events. Safety events were combined into one database for the intention-to-treat population of 2016 patients (pramlintide, *n* = 1434; pooled comparators, *n* = 582). The primary analysis compared major adverse cardiovascular events (MACE) for pramlintide and control treatments, an approach that has been applied to other existing diabetes treatments [17–19]. The protocol of each trial was approved by the respective institutional review board, all participants provided written informed consent prior to study entry, and the studies were conducted in accordance with the principles defined by the Declaration of Helsinki.

**Table 1:** Phase 3 and 4 clinical trials with mealtime pramlintide as adjunct to insulin in patients with type 2 diabetes

Reference	Patient population (N^a^)	Length (weeks)	Comparator	Study treatments	Background therapy
Ratner et al., 2002 [13]	Type 2 diabetes^b^ requiring insulin^c^; mean age 55.5 to 57.5 y between groups; baseline HbA1c 9.0 % to 9.3 % between groups^d^ (538)	52	Placebo	Pramlintide (30, 75, 150 mcg TID) or placebo	Insulin with or without SFU, metformin
Hollander et al., 2003 [12]	Type 2 diabetes^b^ requiring insulin^e^; mean age 56.4 to 57.0 y between groups; baseline HbA1c 9.0 % to 9.3 % between groups^d^ (656)	52	Placebo	Pramlintide (60 mcg TID, 90 mcg BID, 120 mcg BID) or placebo	Insulin with or without SFU, metformin
AstraZeneca (data on file) [14]	Type 2 diabetes^b^ requiring insulin^e^; mean age 57.8 y; HbA1c 9.3 % to 9.5 % between groups^d^ (499)	26	Placebo	Pramlintide (90 mcg BID, 120 mcg BID, 90 mcg TID) or placebo	Insulin with or without SFU, metformin
Riddle et al., 2007 [15]	Type 2 diabetes not controlled by insulin glargine^f^; mean age 55 y; baseline HbA1c 8.5 %^g^ (211)	16	Placebo	Pramlintide (60 or 120 mcg BID/TID) versus placebo	Insulin glargine with or without metformin, SFU, TZD
Riddle et al., 2009 [16] (phase 1 only)	Type 2 diabetes; mean age 55 y and 54 y; baseline HbA1c 8.2 % and 8.3 %; insulin-naïve or used <50 U/d of basal insulin for <6 months^g^ (112)	24	Rapid-acting insulin	Pramlintide (60 or 120 mcg BID/TID) versus rapid-acting insulin	Insulin glargine or detemir with or without metformin, SFU, TZD

*BID* twice daily, *SFU* sulfonylurea, *TID* three times daily, *TZD* thiazolidinedione

^a^Intent-to-treat population

^b^Free of symptoms of severe hypoglycemia or hyperglycemia for the prior 2 weeks

^c^On a stable insulin dose (±10 %) during the prior week

^d^Excluded patients with heart disease, blood pressure >150/95 mm Hg at screening, or hypertension poorly controlled by treatment

^e^On a stable total daily insulin dose (±10 %) during the prior 2 months

^f^On a stable insulin dose (±10 %) during the prior 1 month

^g^Excluded patients who had experienced recurrent severe hypoglycemia requiring assistance during the past 6 months or who had a history of hypoglycemia unawareness

**Table 2:** Demographics and baseline characteristics

Variable	Pramlintide (*n* = 1434)	Pooled comparator (*n* = 582)
Sex		
Male	738 (51.5)	315 (54.1)
Female	696 (48.5)	267 (45.9)
Age (years)	57.2 ± 10.1	55.7 ± 10.2
Race		
White	1184 (82.6)	467 (80.2)
Black	136 (9.5)	57 (9.8)
Asian	7 (0.5)	3 (0.5)
Hispanic	93 (6.5)	47 (8.1)
Other	14 (1.0)	8 (1.4)
Body weight (kg)	93.6 ± 19.9	94.4 ± 19.6
Body mass index (kg/m^2^)	32.4 ± 6.2	32.6 ± 6.4
HbA1c (%)	9.1 ± 1.2	9.0 ± 1.3
Duration of diabetes (years)	12.6 ± 7.3	12.0 ± 7.0

Data are shown as n (%) or mean ± standard deviation

*HbA1c* glycated hemoglobin

### Cardiovascular events

Cardiovascular safety of pramlintide was assessed using standardized medical definitions (queries) of AEs listed in the standardized *Medical Dictionary for Regulatory Activities* (MedDRA) version 12.0, which had been reported by investigators and listed in clinical study reports submitted previously to regulatory authorities or a clinical database. The primary analysis compared the frequency of investigator-reported MACE in the pramlintide and control treatment groups. As widely accepted, MACE included cardiovascular mortality, myocardial infarction, stroke, hospitalization for acute coronary syndrome, and urgent revascularization procedures [20].

There is a theoretical consideration of type I errors (false negatives) if the criteria that constitute the composite end point are too broad, and a consideration of type II errors (false positives) if criteria are too narrow; thus, secondary analyses were performed to confirm the findings of the primary analysis. The secondary analyses included three supplemental, widely accepted definitions of MACE. First, the narrower “subset MACE” category comprised all terms for cardiovascular mortality, myocardial infarction, and stroke. Second, the standardized MedDRA terms for MACE (“SMQ MACE”) category encompassed all MedDRA query terms for myocardial infarction, central nervous system hemorrhages, and cerebrovascular accidents [21]; the analysis was done as described in an identical exenatide analysis [22]. Third, a broader set of cardiovascular events (“broad CV”) was analyzed, which comprised all query terms for cardiovascular mortality, myocardial infarction, stroke, hospitalization for acute coronary syndrome, urgent revascularization procedures, arrhythmia, heart failure, or mechanical-related events (i.e. all SMQ MACE plus all preferred terms under arrhythmia, heart failure, and mechanical-related events).

### Statistical analysis

Pooled investigator-reported AE data from the pramlintide treatment group were compared with pooled data on investigator-reported AEs from the placebo and rapid-acting insulin treatment groups. Relative risks (RRs) and hazard ratios (HRs) of cardiovascular events with pramlintide versus control were calculated. The RR corresponding to incidence rate was based on the Mantel-Haenszel method stratified by study, and the HR used the Cox proportional hazard model and the Andersen-Gill model. The threshold for HR and RR point estimates was 1.3, as widely used [20]. The upper limit of the two-sided 95 % confidence interval (CI) for the estimated risk ratio was defined as 1.8, as widely used [20]. Kaplan-Meier survival curves weighted according to the number of patients in each study were generated to show the time to first event and proportion of patients risk-free over time. *P*-values using the log-rank test were provided for Kaplan-Meier survival curves. The event rate per 1000 patient-years was calculated based on the Exact method, and the corresponding RR was calculated using the log-normal approximation.

## Results

The number and incidence of investigator-reported cardiovascular events in the pramlintide and pooled comparator treatment groups are listed in Table 3. The incidence of MACE in the pooled data was 4.6 %, with no difference observed between the pramlintide (4.7 %) and comparator (4.5 %) groups. The incidence of fatal events was 0.21 % (*n* = 3/1434) in the pramlintide group and 0.69 % (*n* = 4/582) in the pooled comparator group.

**Table 3:** Comparative incidence of investigator-reported CV events in five pooled clinical trials with pramlintide as adjunct to insulin

Event definition, n (%)	Pramlintide (*n* = 1434)	Pooled comparator (*n* = 582)	Risk ratio (95 % CI)
Primary MACE^a^			
Incidence, n (%)	67 (4.7)	26 (4.5)	
Event rate per 1000 patient-years	95.12	91.98	1.034 (0.694–1.540)
Subset MACE^b^			
Incidence, n (%)	32 (2.2)	12 (2.1)	
Event rate per 1000 patient-years	37.63	36.23	1.039 (0.551–1.958)
SMQ MACE^c^			
Incidence, n (%)	49 (3.4)	15 (2.6)	
Event rate per 1000 patient-years	60.63	50.17	1.208 (0.712–2.051)
Broad CV^d^			
Incidence, n (%)	127 (8.9)	43 (7.4)	
Event rate per 1000 patient-years	190.24	189.53	1.004 (0.760–1.326)

Overall incidence in the pooled population was 4.6 % for primary MACE, 2.2 % for subset MACE, 3.2 % for SMQ MACE, and 8.4 % for broad CV

*CI* confidence interval, *CV* cardiovascular, *MACE* major adverse cardiovascular events, *SMQ MACE* standardized *Medical Dictionary for Regulatory Activities* query for MACE

^a^Primary MACE included CV mortality, myocardial infarction, stroke, hospitalization for acute coronary syndrome, and urgent revascularization procedures

^b^Subset MACE included CV mortality, myocardial infarction, and stroke only

^c^SMQ MACE included myocardial infarction, central nervous system hemorrhages, and cerebrovascular accidents

^d^Broad CV included CV mortality, myocardial infarction, stroke, hospitalization for acute coronary syndrome, urgent revascularization procedures, arrhythmia, heart failure, or mechanical-related events

**Fig. 1:**
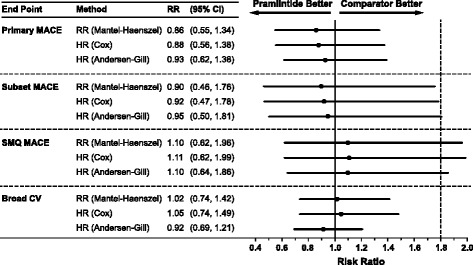
Risk assessment of cardiovascular end point by statistical method. CI: confidence interval; CV: cardiovascular; HR: hazard ratio; MACE: major adverse cardiovascular events; RR: relative risk; SMQ MACE: standardized *Medical Dictionary for Regulatory Activities* query for MACE

More detailed analyses were conducted on each of the cardiovascular event categories listed in Table 3. There was no difference between the pramlintide and pooled comparator groups in the incidence of cardiovascular events by any of the measures (Fig. 1). Figure 1 depicts the RR and HR point estimates (and 95 % CI) of cardiovascular events according to each category in the pramlintide versus comparator treatment groups. The risk ratios ranged from 0.86 to 0.93 for MACE for pramlintide treatment versus comparator; combined with the 95 % CI shown, the data showed that pramlintide had a similar risk of cardiovascular events as the comparator treatment (Fig. 1). The upper limit of the two-sided 95 % CI for the estimated risk ratio of primary MACE did not exceed the threshold of 1.8 (0.55–1.34 by Mantel-Haenszel analysis). For each category of cardiovascular events listed (Table 3; Fig. 1), the upper limit of the two-sided 95 % CI was between 1.3 and 1.8 and did not exceed the threshold of 1.8 (Fig. 1). Estimates of RR and HR were consistent for each secondary end point, regardless of the analysis method used.

### Event rates

**Fig. 2:**
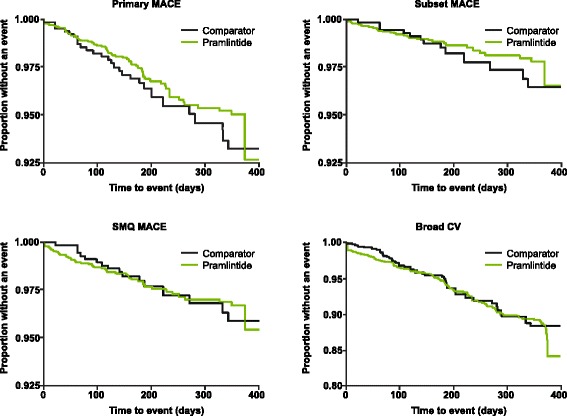
Weighted Kaplan-Meier plots for time to first event for cardiovascular adverse events. *P*-values using the log-rank test were *P* < 0.0001 for primary MACE, *P* < 0.0001 for subset MACE, *P* = 0.3993 for SMQ MACE, and *P* = 0.3733 for broad CV. CV: cardiovascular; MACE: major adverse cardiovascular events; SMQ MACE: standardized *Medical Dictionary for Regulatory Activities* query for MACE

Figure 2 depicts the proportion of patients without an event over time, meaning that a treatment that has fewer cardiovascular events would show a larger proportion of event-free patients. The analyses for Fig. 2 used cardiovascular event rates that were weighted by the number of patients in each study. The data showed no excess risk of cardiovascular events in any analysis category with pramlintide versus comparator treatments.

## Discussion

The results indicate that no excess cardiovascular risk was associated with mealtime pramlintide treatment in patients with type 2 diabetes who had inadequate glycemic control. The primary analysis of MACE showed that both the point estimate and range of the 95 % CI of the estimated risk ratio were consistent with the absence of an increased risk of cardiovascular events. Some point estimates showed possibly less risk of a cardiovascular event in the pramlintide treatment group—when events were in the primary analysis category, MACE. The weighted Kaplan-Meier analysis of primary MACE (Fig. 2) was also consistent with the absence of an increased risk of cardiovascular events. The incidence of fatal cardiovascular events was lower in the pramlintide treatment group compared to the comparator treatment group. The secondary analyses were supportive of the primary analysis because no differences between treatment groups in the risk of cardiovascular events were found. The consistency between the various statistical approaches applied to this data set of investigator-reported cardiovascular AEs was reassuring.

The patients enrolled in the first three pramlintide studies reported here were diagnosed with type 2 diabetes with a mean duration of ≥12 years, had mean baseline HbA1c levels ≥9.0 % while using insulin, and were overweight (mean weight, 94 kg; mean body mass index, 32–33 kg/m^2^), corresponding to an increased risk of cardiovascular events, which may be as high as 2–3 times that of the general population [23]. Three of the five pramlintide studies were conducted under the principle of “insulin-equipoise” (i.e. at the time, the instructions for patients were not to adjust the dose of insulin when initiating pramlintide; currently, the US label for pramlintide mandates a reduction by 50 % of mealtime insulin at initiation and subsequent slow up-titration of insulin based on blood glucose levels) [24]. The MACE frequency observed in the pramlintide studies (4 % in 1 year) was similar to the ORIGIN cardiovascular outcome study with insulin glargine (MACE frequency approximately 3 % per year) [25], and comparable as well to the MACE frequency in the placebo treatment group in recent cardiovascular outcome studies (between 3 and 4 % per year) [25–28]. In summary, the pooled pramlintide studies were found to have a frequency of cardiovascular events similar to that in cardiovascular outcome studies, supporting the robustness of the findings.

The present retrospective analysis has limitations of a relatively small sample size of approximately 2000 patients, who were generally of middle age, and relatively short observation periods of 16 to 52 weeks. Several existing diabetes treatments presented systematic retrospective analyses to estimate their cardiovascular safety signals, recognizing that cardiovascular events are often not adjudicated [17–19, 22, 29]. These analyses found that glucose-lowering therapies including dipeptidyl peptidase-4 inhibitors and glucagon-like peptide-1 receptor agonists were also not associated with increased cardiovascular risk, while reporting low MACE rates [17–19, 22, 29]. The several-fold lower MACE rates observed in systematic reviews of early clinical development studies were not replicated in formal cardiovascular outcome studies, likely because of differences in inherent baseline cardiovascular risk in study populations [26–28]; however, the initial finding of no excess cardiovascular risk [17–19, 22, 29] was confirmed. Therefore, a potential limitation of retrospective analyses is that too few cardiovascular events might have occurred, because the populations enrolled in pivotal clinical trials is generally healthier with fewer CV risk factors; however, the pooled pramlintide studies analyzed here showed a sufficiently high frequency of cardiovascular events (4 % over 52 weeks) for these analyses.

It has been recognized that the risk of hypoglycemia is one of the most significant factors that limit optimization of insulin therapy [30, 31]. In general, addition of insulin to the treatment regimen, longer duration of insulin use, and intensive treatment increase the risk of hypoglycemia [25, 30]. It is of interest that the incidence of severe hypoglycemia was similar between pramlintide and control treatment groups in the studies included in the present analysis [12–16], although the risk of severe hypoglycemia was generally higher during the first weeks following initiation of pramlintide therapy [12, 13]. The relationship between hypoglycemia and increased cardiovascular risk is not clear at present [30, 32, 33]. Several smaller studies showed associations between low blood glucose and cardiac ischemia [34] and angina (a case study) [35]. More recently, a connection between higher hypoglycemia rates and higher mortality rates in the ACCORD trial was made [36]. However, subsequent post hoc analyses showed that the excess mortality in the intensive treatment group was not directly explained by a higher rate of severe hypoglycemia [37]. Analyses of the ADVANCE trial showed an association between severe hypoglycemia and increased risk of macrovascular events, microvascular events, and cardiovascular death, indicating that hypoglycemia is likely “a marker of vulnerability to a wide range of adverse clinical outcomes” [38]. However, the incidence of severe hypoglycemia was similar for the pramlintide and insulin groups in the studies included in the present analysis.

One recent overview of prospective cardiovascular outcome trials in type 2 diabetes describes evidence that, in patients with type 2 diabetes, glucose-lowering per se is likely to reduce cardiovascular events [32]. For example, reduction in cardiovascular events has been demonstrated with a metformin-based glucose-lowering treatment strategy [39] and in a meta-analysis describing UK Prospective Diabetes Study data combined with three large trials [30]. Published prospective cardiovascular outcome studies of pharmaceutical agents have been conducted under the guiding principle of glycemic equipoise to better evaluate the safety of the pharmaceutical agent under study, although glycemic equipoise does not apply to all ongoing cardiovascular outcome studies [40, 41]. The glycemic-equipoise design has the potential to bring out pleiotropic actions of a drug, in addition to glucose lowering, although treatment differences in HbA1c can still occur. The EMPA-REG outcome trial in 7020 patients with type 2 diabetes, which evaluated the oral sodium-glucose cotransporter 2 inhibitor empagliflozin, is the only study thus far showing a risk reduction (of 14 %) in MACE (risk ratio of 0.86 [95 % CI, 0.74 to 0.99]), while an additional benefit was observed in hospitalizations for heart failure [28].

Prior to this analysis, pramlintide as a pharmaceutical agent was considered neutral with regard to risk of cardiovascular events, and that view has not changed based on the current findings. The efficacy of pramlintide in improving post-meal hyperglycemia and long-term glycemic control (−0.6 % HbA1c reduction vs. placebo [12, 13]) in inadequately controlled diabetes in combination with insulin, without weight gain (or with weight loss), can plausibly help reduce the hyperglycemia-mediated cardiovascular risk, but that has not been formally tested. The mechanism of action of pramlintide includes delayed gastric emptying, modulation of satiety leading to decreased food intake, and reduction of post-meal glucagon secretion, resulting in more efficient insulin action with regard to glucose disposal following ingestion of exogenous glucose (reviewed in Hinshaw et al. [4]). Furthermore, pramlintide has been associated with beneficial effects on body weight, lipids (total cholesterol, low-density lipoprotein cholesterol, and triglycerides) [42], as well as other emerging cardiovascular risk markers [42–45]. Combined, these features of pramlintide are consistent with the absence of an excess risk of cardiovascular events associated with its use.

## Conclusions

The findings of the current retrospective pooled analysis add to the body of safety data on pramlintide use in patients with type 2 diabetes inadequately controlled on insulin. The findings have inherent limitations mostly attributed to the short observation period (16 to 52 weeks) and relatively small sample size, but these are less likely to have influenced the results because the cardiovascular event rate in the pooled assessment was sufficient for robust statistical analyses. The cardiovascular events analyzed were investigator-reported MACE that had occurred in the clinical trials, and had been categorized according to standardized regulatory medical definitions. In conclusion, this new analysis of pooled clinical trial data showed that there was no increased risk of MACE associated with pramlintide therapy versus comparator treatment in patients with type 2 diabetes using insulin.

## Abbreviations

AEs: Adverse events
BID: Twice daily
CI: Confidence interval
CV: Cardiovascular
HbA1c: Glycated hemoglobin
HR: Hazard ratio
MACE: Major adverse cardiovascular events
MedDRA: *Medical Dictionary for Regulatory Activities*
RR: Relative risk
SFU: Sulfonylurea
SMQ MACE: Standardized MedDRA query for MACE
TID: Three times daily
TZD: Thiazolidinedione
